# Letter to the Editor: Deep cervical lymphovenous anastomosis (LVA) for Alzheimer’s disease: microsurgical procedure in a prospective cohort study

**DOI:** 10.1097/JS9.0000000000002937

**Published:** 2025-07-03

**Authors:** Xueting Wang, Yue Tian

**Affiliations:** Department of Anesthesiology, Shengjing Hospital of China Medical University, Shenyang, Liaoning, China.

*Dear Editor,*

We have read with great interest the recent publication by Chen *et al*^[1]^. This innovative study presents a new surgical technique designed to tackle the increasing challenge of Alzheimer’s disease (AD) by improving brain lymphatic drainage, marking an exciting step forward in the treatment of neurodegenerative disorders. This pioneering study presented a novel surgical technique for the treatment of AD; however, lymphovenous anastomosis (LVA) is not a new surgical procedure. It was first reported in 1962 for the treatment of chyluria and later used for the treatment of lymphedema^[2]^. While there have been a few case reports, this article represents the first prospective study on the subject^[3,4]^. It should be noted that our article is compliant with the TITAN Guidelines 2025^[5]^.

The author’s assessment of the safety and effectiveness of the procedure provided useful insights. This study collected data through quantitative analyses of neuropsychological assessments conducted before and after surgery along with cerebrospinal fluid (CSF) biomarkers and imaging examinations. These components are crucial to understanding the effects of the intervention. The author’s modification of the traditional LVA technique, which involves the formation of lymphatic flaps instead of directly connecting lymphatic vessels to veins, is noteworthy. This innovation could alleviate some of the technical challenges associated with microsurgery while also reducing the risk of damage to fragile lymphatic structures, which would be highly beneficial for primary care hospitals. Additionally, the authors highlight the value of routine intraoperative electrophysiological monitoring to prevent nerve injuries. The results indicated that 60% of healthcare personnel reported an improvement in patients’ overall condition, along with a significant increase in postoperative MMSE scores, providing preliminary evidence of LVA’s potential benefits for cognitive function. This offers renewed hope to patients who have not found relief through traditional pharmacological treatment.

While this study is pioneering, several areas warrant further investigation to enhance its clinical implications and understanding. Although the study observed trends toward decreased biomarker levels after LVA, these changes were not statistically significant. A more in-depth statistical analysis, potentially involving larger cohorts or multi-center designs, may provide clearer insights into the mechanisms by which LVA affects AD pathology. We believe that reliance on CSF may limit the study’s broader applicability. Currently, both domestic and international efforts are exploring the accessibility of blood biomarkers for AD and their potential to facilitate large-scale screening. Studies conducted on different analytical platforms have shown that plasma Aβ can accurately detect cerebral amyloidosis, and that the Aβ42/40 ratio is more strongly correlated with the brain Aβ burden. The diagnostic and predictive accuracy of this ratio exceeds that of Aβ42 or Aβ40 alone, while various combinations of biomarkers can often enhance the overall accuracy of Aβ measurements^[6]^. Recent studies have confirmed that p-tau217 is a reliable target in plasma samples for identifying AD pathology^[7]^. Other blood biomarkers, including the astrocyte activation marker GFAP and the axonal injury marker NFL, may contribute to tracking the course of Alzheimer’s disease and its response to treatment^[6]^. We believe that subsequent follow-up of biomarkers related to this surgical procedure may appropriately consider the application of plasma biomarkers.

The article identifies potential confounding factors, and the authors acknowledge that the differences in postoperative cognitive rehabilitation and the natural progression of Alzheimer’s disease may affect the outcomes. Therefore, it is essential to quantify and control for these confounding factors. Additionally, all patients underwent surgery under general anesthesia, which is associated with the development of Alzheimer’s disease symptoms. The incidence of perioperative neurocognitive disorders (PND) is higher in patients with AD, and the consequences are more severe. The excessive deposition of Aβ and tau proteins, along with neuroinflammation, represents a common pathological intersection between AD and PND^[8,9]^. The impact of general anesthesia on the vulnerable brain should be an important consideration when evaluating the prognosis of this new surgical method.

This study indicates that LVA may mainly work by improving glymphatic drainage instead of directly targeting existing Aβ plaques. It is important to continue investigating how this process works at the biological level. Investigating whether LVA can help to clear soluble Aβ and how this might affect cognitive and functional outcomes will strengthen the case for this surgical approach.

The study of LVA for Alzheimer’s disease lays a crucial groundwork for exploring surgical therapies for AD and raises essential questions about the future of AD treatment strategies. We commend the authors for their innovative work and look forward to more extensive studies that could solidify the role of surgical interventions in managing Alzheimer’s disease.

Thank you for considering our thoughts on this important research.

## Data Availability

This is a comment on a published paper; no data were presented in our comment, so the data statement was not applicable.
